# An Assessment of the Clinical and Economic Impact of Establishing Ileocolic Anastomoses in Right-Colon Resection Surgeries Using Mechanical Staplers Compared to Hand-Sewn Technique

**DOI:** 10.1155/2015/749186

**Published:** 2015-08-27

**Authors:** S. Roy, S. Ghosh, A. Yoo

**Affiliations:** ^1^Global Health Economics and Market Access, Ethicon, Somerville, NJ 08876, USA; ^2^Global Health Economics and Market Access, Ethicon, Cincinnati, OH 45242, USA; ^3^Medical Devices Epidemiology, Johnson & Johnson, New Brunswick, NJ 08901, USA

## Abstract

*Purpose*. To estimate and compare clinical outcomes and costs associated with mechanical stapling versus hand-sewn sutured technique in creation of ileocolic anastomoses after right sided colon surgery. *Methods*. A previously conducted meta-analysis was updated for estimates of anastomotic leak rates and other clinical outcomes. A value analysis model was developed to estimate cost savings due to improved outcomes in a hypothetical cohort of 100 patients who underwent right colon surgery involving either mechanical stapling or hand-sewn anastomoses. Cost data were obtained from publicly available literature. *Results*. Findings from the updated meta-analysis reported that the mechanical stapling group had lower anastomotic leaks 2.4%  (*n* = 11/457) compared to the hand-sewn group 6.1% leaks (*n* = 44/715). Utilizing this data, the value analysis model estimated total potential cost savings for a hospital to be around $1,130,656 for the 100-patient cohort using mechanical stapling instead of hand-sewn suturing, after accounting for incremental supplies cost of $49,400. These savings were attributed to lower index surgery costs, reduced OR time costs, and reduced reoperation costs driven by lower anastomotic leak rates associated with mechanical stapling. *Conclusion*. Mechanical stapling can be considered as a clinically and economically favorable option compared to suturing for establishing anastomoses in patients undergoing right colon surgery.

## 1. Introduction

Ileocolic resection is the most frequently performed surgical procedure for the treatment of right-sided colorectal cancer and Crohn's disease [1]. Surgical treatment for these conditions includes resection of the diseased bowel and formation of an ileocolic anastomosis. Anastomotic leak is one of the most dreaded postoperative complications in patients particularly after resection of the colon and the rectum. Further, reoperations and complications such as leaks are considered a quality indicator in colorectal surgery [2]. The prevalence of anastomotic leaks after colon and rectal resection varies by anatomic location with lower frequencies in right sided anastomoses. The reported range for radiologically identified leaks is between 0.5% and 21% while the incidence of clinically significant anastomotic leaks after colorectal surgeries is between 1% and 12% and up to 10% to 14% in low colorectal resections [2]. Overall, patients with anastomotic leaks after colorectal surgery have significantly greater chances of morbidity (56%) and mortality rates of up to 32 % [3]. In addition to the clinical complications there is a significant economic burden to be considered as multiple reoperations, radiologic interventions, and stoma creation are often necessary to control leaks, and hospital length of stay for these patients is reported to be longer thus resulting in an increase in health care cost compared to patients with no leaks. Therefore, anastomotic leaks can impose a significant burden on patients and health care providers.

Over the years, various techniques of colorectal anastomosis have been developed in search of one with lower rate of postoperative complications [4]. The introduction of stapling devices has helped to revolutionize the technical aspects of surgery that has allowed minimally invasive procedures to be developed and performed more quickly than manual sutures. Findings from a recent Cochrane systematic review and meta-analysis reported that stapled colorectal anastomosis resulted in significant reduction in anastomotic leaks compared to hand-sewn technique in right colon resections. Leak rates after colorectal surgery using stapled and hand-sewn anastomosis have been reported in the literature to be around 8% and 27%, respectively [5]. In addition, stapled ileocolic anastomoses took on an average 8.7 minutes compared to 22.4 minutes for hand-sewn technique [1].

A number of benefits conferred by the use of stapling techniques include uniformity of surgical technique, minimal tissue manipulation and trauma, less bleeding and edema at the site of anastomosis, a quicker return of gastrointestinal functions, and more rapid patient recovery which together have made the technique a desirable alternative for anastomosis compared to hand-sewing with sutures [6]. Conversely, stapling techniques have also been criticized on the grounds of expense and low improvements in anastomotic outcomes. Despite comparable results in terms of mortality, anastomotic leaks, and wound infection, the rate of stricture at the anastomotic site has been reported as considerably higher with staples than with sutures: around 8% versus 2%, respectively, for colorectal anastomosis [7].

Therefore, there is an ongoing search for an ideal method of establishing an anastomosis that will not only lower the incidence of dangerous complications but also avoid the need for reoperations. Additionally, there is limited evidence in the literature outlining the economic value of using one technique over the other for ileocolic anastomosis.

The main objectives of this study were (1) to update earlier estimates of anastomotic leak rates following ileocolic anastomosis performed using mechanical stapling and hand-sewn techniques and (2) to develop a value analysis model to estimate and compare the treatment costs associated with the two surgical options for patients undergoing elective or emergency ileocolic anastomosis from a hospital perspective.

## 2. Methods

### 2.1. Literature Review and Meta-Analysis

A comprehensive systematic search of literature was conducted using MEDLINE, EMBASE, Scopus, Cochrane library, and trial registry databases to identify studies from a period of January 1990 to December 2013 comparing clinical outcomes associated with mechanical stapling and hand-sewn suturing for ileocolic anastomosis in adults. Studies that used mechanical stapler (side-to-side or functional end-to-end) or manual suturing (hand-sewn) for ileocolic anastomosis were reviewed. The primary outcome of interest was overall anastomotic leak rates for each technique while some of the secondary outcomes of interest were rates of reoperation, anastomosis time, and length of hospital stay. The review was conducted and reported according to QUORUM guidelines. The titles and abstracts of articles found in the original search were screened by two independent reviewers. Following that, full texts of eligible studies were obtained and another reviewer independently determined the eligibility of each publication by applying a set of criteria described in Table 1. Cited references from included trials and reviews of similar trials were also searched. All studies that met the inclusion criteria were included in the review. Two independent reviewers extracted study characteristics, baseline, and outcomes data. The methodological quality of publications was assessed using the criteria previously reported in an earlier Cochrane review [1]. A third reviewer checked the resulting extractions and resolved any discrepancies. Parameters that were extracted from each study included study type, country, procedure, reason for right colon resection surgery, anastomosis location, sample size, number of patients with anastomotic leaks in each group, methods of anastomotic leak diagnosis, time required for anastomosis, nonleak complication rate, and overall complication rate. Meta-analysis was conducted to pool results for the outcomes of interest using the RevMan 5 software. Outcomes were summarized as odds ratios (OR) using the Mantel-Haenszel fixed-effects modeling with Chi-square test for heterogeneity.

### 2.2. Value Analysis

A cohort approach was used to develop a value analysis model to understand the financial implications for a hospital utilizing mechanical stapling versus hand-sewn sutured anastomoses. The model focused on estimating cost savings due to reduced leak rates, lower number of reoperations/readmission rates, and reduced operating room time associated with each technique using a hospital perspective. The target population evaluated in the model consisted of patients who underwent elective or emergency open right colon surgery using either mechanical stapling or hand-sewn sutured anastomosis. The model leveraged the leak rates data from the review and meta-analysis described above and utilized those rates to calculate differences in incidence and costs related to leaks, both in the index procedure and for readmission. In addition, for other outcomes, such as risk of reoperation and anastomosis time, the model included data from the literature that were identified during the review but not included in the meta-analysis, primarily as they were not randomized controlled trials.

All cost data were obtained from publicly available literature.  Table 2 lists the cost inputs used for calculating costs related to ileocolic anastomoses in a hypothetical cohort of 100 patients compared between mechanical stapling with manual suturing. The cost of colorectal surgery with and without leaks was based on the findings of a recent retrospective analysis conducted in 6,174 patients in the United States, where anastomoses were established using mechanical stapling or hand-sewn suturing [8]. An average cost of $44,308 for a colorectal surgery without a leak was used in the model. Furthermore, as patients with anastomotic leaks had 1.3 times higher 30-day readmission risk, the incremental cost of readmissions of $6,409 was used in the analysis [9]. Direct cost for anesthetic services (i.e., cost per 15-minute block of anesthesia time) was obtained from published results of the American Society of Anesthesiologists survey [10].

As mechanical stapling was expected to result in a more favorable cost outcome, a scenario analysis was conducted by forcing model inputs to be significantly less favorable to the mechanical stapling option in order to examine the level of robustness of the data.

## 3. Results

The literature review identified four new studies in addition to those that were already included in an earlier Cochrane review. Overall twelve studies that met the study inclusion criteria were identified for the review, of which eight were randomized control trials (RCTs), three were retrospective assessments, and one was a prospective study. There were no significant differences between most of the patient baseline characteristics. Follow-up duration ranged from 30 days after discharge to a median of 87 months [13, 15].

**Figure 1:**
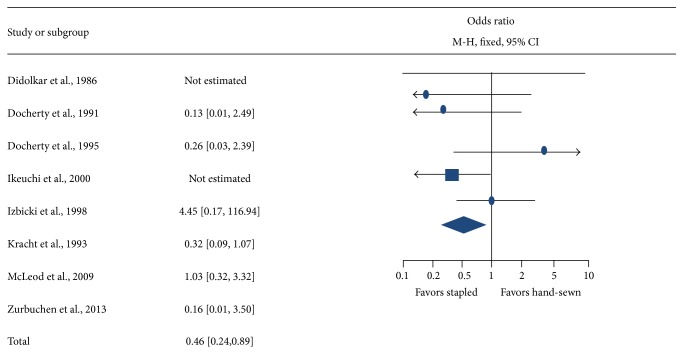
Forrest plot of comparison using data from all studies for anastomotic leak rates.

Eight RCTs with a total of 1,172 patients with ileocolic anastomosis were included in the pooled meta-analysis. Details of the RCTs included in the analysis are presented in Table 3. Of the RCTs included, 2 studies were from Germany, 2 were from Scotland, 1 was from France, 1 was from Japan, 1 was from US, and 1 was a global study with patients from US, UK, and Canada. The nonrandomized studies were conducted in UK and Italy. The main findings from the study demonstrated that the mechanical stapling group had lower (2.4%) anastomotic leaks (*n* = 11/457) compared to 6.1% leaks reported (*n* = 44/715) in the hand-sewn group (Table 4). Overall, the mechanical stapling group had significantly lower odds (0.46; 95% CI = 0.24–0.89; *P* = 0.02) of anastomotic leaks compared with the hand-sewn anastomosis group (Figure 1).

The rate of reoperation, when reported, was also lower for the mechanical stapling group compared to the hand-sutured group, with the difference ranging from 4.3% to 26.1% in one study [18]. Furthermore, mechanical stapling was faster and saved on average 13.6 minutes per patient compared to hand-sewn technique in a study that captured and reported anastomosis time [6]. Tables 2 and 5 report economic and clinical estimates from the meta-analysis and from other pieces of published literature that were included in the value analysis. Inputs used in the scenario analysis are described in Tables 2 and 5.

**Figure 2:**
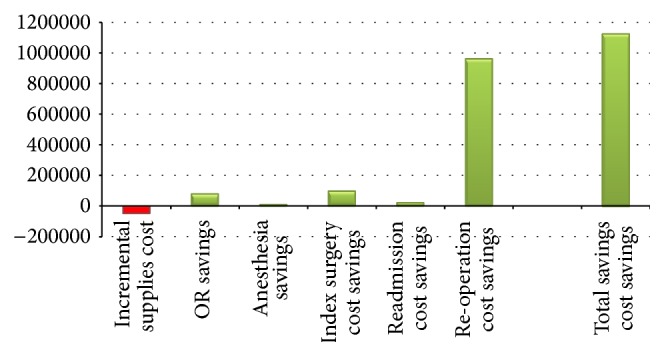
Potential cost savings using mechanical staplers.

Findings from the value analysis model demonstrated that with the included inputs and assumptions ileocolic anastomosis established in a cohort of 100 patients using mechanical stapling instead of hand-sewn suturing could result in significant savings for a hospital. The savings were estimated at around $1,130,656 for the cohort of 100 patients or about $11,000 per patient procedure. The savings were net of incremental supplies cost of about $50,000 that reduced the overall savings by about 4%. The cost savings were primarily realized through avoidance of incremental costs, both in the index procedure [$96,516 (9%)] and in readmissions [$25,636 (2%)] that were made possible with reduced anastomotic leak rates with mechanical stapling compared to hand suturing. Large savings of $965,914 (85%) could be achieved due to lower rates of reoperations/readmissions for patients who had mechanical stapling. Furthermore, from a hospital perspective, as mechanical stapling is faster compared to hand-sewn suturing, the requirement for anesthetic services and OR time was substantially lower, leading to cost savings of about $7,162 (1%) and $84,827 (8%), respectively (Figure 2). In addition, owing to the shorter time taken for the anastomosis, the collective gain in operating room time could be close to 23 hours for the cohort of 100 procedures, thus freeing up the operating room for potential additional patient care utilization.  Table 6 represents key results from the value analysis model.

Results of the model were robust to the effect of conservative assumptions employed in a scenario analysis.  Table 6 presents the corresponding results which show that in spite of enforcing significant reductions in potentially better outcomes with stapling the hypothetical hospital could retain an overall net saving of about $153,907 which translates to a saving of about $1,539 per person.

## 4. Discussion

Anastomotic leaks are among the most prevalent and detrimental complications that occur after colorectal surgery. Postoperative anastomotic leaks remain a significant complication and are associated with high morbidity, mortality, reoperation, and duration of hospitalization [19–23]. In cases of surgery for malignant pathology, anastomotic leakage is related to diminished five-year disease-specific survival and higher local recurrence rates [11, 21, 24]. It is therefore imperative for health care providers to find optimal techniques to prevent postoperative anastomotic leaks which can possibly help to ease the associated clinical and economic burden. It has been documented that anastomotic leaks are the strongest indicators of hospital costs in colorectal surgeries and impose a significant economic burden on patients and health care providers due to additional readmission rates, reoperations, postoperative infections, and longer durations of hospital stay [25]. Patients with anastomotic leaks have a 1.3-fold greater chance of readmission within a 30-day period compared to those without leaks which leads to a significant increase in the overall cost of care. It has been reported that patients with leaks spend approximately 7 days more in a hospital with average incremental costs of $24,129 compared to those without leaks [9]. The total burden of leaks in terms of length of stay per 1,000 patients was 16,800 and 26,300 days for patients with no leaks and with leaks, respectively [9]. Furthermore, the total cost burden per 1,000 patients was reported to be $44.3 million in patients with no leaks as compared to $72.9 million for those with leaks, which further highlights the negative impact of anastomotic leaks and underscores the importance of cost reductions for patients and hospitals using appropriate anastomotic techniques [9].

In recent years, to inform decision-making by surgeons, evidence has been generated to show how certain anastomotic techniques, such as the stapled side-to-side technique, are more advantageous while considering treatment for specific conditions such as cancer and Crohn's disease as they are simple, uniform, reliable, and safe to perform [24, 26]. This is also supported by results from the meta analysis conducted in the current study which suggests the possibility of clinical benefit from the use of mechanical stapling following a right colon resection due to lower anastomotic leaks compared to hand-sewn technique especially if the operation is performed in patients with colon cancer. The study also estimates potential cost savings from a hospital perspective that can be availed using mechanical stapling technique, where appropriate.

While the present study outlines the advantages of mechanical stapling, there are few potential limitations that need to be considered. The study modeled net cost savings of using mechanical stapling for ileocolic resections by making certain assumptions and utilizing data from published literature for key parameters which makes the findings subject to all general limitations applicable for such assessments. The model arguably presents a conservative assessment of potential benefit of the lesser risk of anastomotic leaks as it does not consider costs associated with mortality. While anastomotic leak rates were found to be lower with stapling, it is also important to mention for fair balance that studies included in the review reported additional outcomes, some of which were better in the hand-sutured group of patients. While these outcomes may or may not have had any direct impact on leak rates, they could potentially somewhat reduce expected savings from reduction in leak rates.

One important consideration relevant to the effectiveness of device use and surgical technique is the level of skill a surgeon possesses. This study does not account for the potential impact of surgeon skills and learning curve upon the surgical outcome. As this is one of the first studies to quantify the financial benefits of mechanical stapling compared to suturing in the establishment of an ileocolic anastomosis using a model built on evidence from literature, future research needs to focus on conducting real-world studies to support this finding.

## 5. Conclusion

In conclusion, the results of this study underscore the potential clinical and economic benefits of mechanical stapling compared to hand-sutured anastomosis in right colon surgery. Such benefits are attributed to cost reduction owing to a meaningful reduction in the risk of anastomotic leaks which likely results in reduced length of inpatient stay, lower rate of readmission and reoperation postdischarge, and shortened anastomosis time.

## Figures and Tables

**Table 1:** Study inclusion criteria for the systematic review and meta-analysis.

Criterion	Included
Population	Age: ≥18 years
	Race: any
	Gender: male or female
	Studies conducted in humans only
	Patients receiving elective or emergency stapled and hand-sewn ileocolic anastomoses

Type of studies	RCTs comparing mechanical stapling and hand-sewn suturing related to colon resection and colonic anastomosis, meta-analysis, systematic reviews, comparative prospective nonrandomized observational studies, and comparative retrospective reviews

Language	English only

Country	Any

Sample size	Any

Intervention	Mechanical stapling versus hand-sewn suturing

Primary outcome	Overall anastomotic leak rates

**Table 2:** Cost inputs included in value analysis model.

Parameters	Base case	Source	Scenario analysis	Source
Cost per linear stapler	$300.00	Assumption		

Cost per stapler reload	$100.00	Assumption		

Cost per 15-minute block of anesthesia time [10]	$71.62	Byrd and Singh, 2010	$35.81	50% reduction; assumption
Incremental index hospitalization costs for patients with leaks [9]	$24,129.00	Hammond et al., 2014	$12,064.50	
Average cost of a colorectal surgery without a leak [9]	$44,308.00	Hammond et al., 2014	$22,154.00	

Number of stapler reloads used per anastomosis	2	Assumption		

Cost per suture strand	$3.00	Assumption		

Charge per minute of OR time [8]	$62.19	Shippert, 2005	$31.10	50% reduction; assumption
Incremental readmission costs for patients with leaks [9]	$6,409.00	Hammond et al., 2014	$3,204.50	

Number of sutures used per anastomosis	2	Assumption		

**Table 3:** Clinical trials included in the meta-analysis.

Study or subgroup	Year	Stapled		Hand-sewn			Odds ratio
		Events	Total *N*	Events	Total *N*	Weight	M-H, fixed, 95% CI
Didolkar et al. [11]	1986	0	22	0	16		Not estimated
Docherty et al. [12]	1991	0	70	4	87	13.6%	0.13 [0.01, 2.49]
Kracht et al. [13]	1993	3	106	26	334	44.5%	0.32 [0.09, 1.07]
Docherty et al. [6]	1995	1	133	4	122	12.9%	0.26 [0.03, 2.39]
Izbicki et al. [14]	1998	1	15	0	21	1.3%	4.45 [0.17, 116.94]
Ikeuchi et al [15]	2000	0	11	0	18		Not estimated
McLeod et al. [16]	2009	6	84	6	86	18.7%	1.03 [0.32, 3.32]
Zurbuchen et al. [17]	2013	0	36	2	31	9.0%	0.16 [0.01, 3.50]

**Table 4:** Postoperative anastomotic leak rates between the two groups as reported in the articles included in the review.

Study or subgroup	Stapled		Hand-sewn			Odds ratio
	Events	Total *N*	Events	Total *N*	Weight	M-H, fixed, 95% CI
Total (95% CI)	—	457	—	715	100%	0.46 [0.24, 0.89]
Total events	11	—	44	—		

Heterogenecity Chi^2^ = 5.39, df = 5 (*P* = 0.37), and *I* ^2^ = 7%.

Test for overall effect: *Z* = 2.31 (*P* = 0.02).

**Table 5:** Clinical inputs included in value analysis model.

Parameters	Base case		Scenario analysis		
	Stapled	Hand-sewn	Stapled	Hand-sewn	Source
Overall leak rate [1]	2.49%	6.14%	2.49%	3.07%	50% reduction for hand-sewn
Reoperation rate [15]	4.3%	26.1%	4.3%	13.1%	

Average time for anastomosis [1]	8.72 min	22.36 min	13.84 min	10.82 min	Most difference found in the literature

**Table 6:** Potential cost savings using mechanical staplers.

Parameters	Base case results	% contribution to savings	Scenario analyses results
Total number of patients using open mechanical staplers	100		100
Potential OR time savings	23 hours		−5 hours
Supplies cost for open mechanical staplers	$50,000	−4%	$50,000
Supplies cost for sutures	$600		$600
Potential savings in OR time cost	$84,827	8%	$ −9,391
Potential savings in anesthesia cost	$7,162	1%	$3,581
Potential savings in index surgery costs through avoided anastomotic leaks	$96,516	9%	$12,065
Potential savings in readmission costs through avoided anastomotic leaks	$25,636	2%	$3,205
Potential savings in reoperation costs	$965,914	85%	$193,848

Net savings using open mechanical staplers	$1,130,656	100%	$153,907
Net savings per patient using open mechanical staplers	$11,307		$1,539

## References

[1] Choy P. Y. G., Bisset I. P., Docherty J. G. (2011). Stapled versus handsewn methods for ileocolic anastomosis. *Cochrane Database of Systematic Reviews*.

[2] Morris A. M., Baldwin L.-M., Matthews B. (2007). Reoperation as a quality indicator in colorectal surgery: a population-based analysis. *Annals of Surgery*.

[3] Choi H.-K., Law W.-L., Ho J. W. C. (2006). Leakage after resection and intraperitoneal anastomosis for colorectal malignancy: analysis of risk factors. *Diseases of the Colon & Rectum*.

[4] Fouda E., El Nakeeb A., Magdy A., Hammad E. A., Othman G., Farid M. (2011). Early detection of anastomotic leakage after elective low anterior Resection. *Journal of Gastrointestinal Surgery*.

[5] Gajda A., Bielecki K. (1999). *The Causes and Prevention of Anastomotic Leak After Colorectal Surgery*.

[6] Docherty J. G., McGregor J. R., Akyol A. M., Murray G. D., Galloway D. J. (1995). Comparison of manually constructed and stapled anastomoses in colorectal surgery. *Annals of Surgery*.

[7] Lustosa S. A., Matos D., Atallah A. N., Castro A. A. (2001). Stapled versus handsewn methods for colorectal anastomosis surgery. *Cochrane Database of Systematic Reviews*.

[8] Shippert R. (2005). A study of time-dependent operating room fees and how to save $100000 by using time-saving products. *American Journal of Cosmetic Surgery*.

[9] Hammond J., Lim S., Wan Y., Gao X., Patkar A. (2014). The burden of gastrointestinal anastomotic leaks: an evaluation of clinical and economic outcomes. *Journal of Gastrointestinal Surgery*.

[10] Byrd J. R., Singh L. (2010). ASA survey results for commercial fees paid for anesthesia services—2010. *American Society of Anesthesiologists*.

[11] Didolkar M. S., Reed W. P., Elias E. G., Schnaper L. A., Brown S. D., Chaudhary S. M. (1986). A prospective randomized study of sutured versus stapled bowel anastomoses in patients with cancer. *Cancer*.

[12] Docherty J. G., Rankin E., Galloway D. J. Anastomotic integrity and local recurrence after colorectal cancer surgery.

[13] Kracht M., Hay J.-M., Fagniez P.-L., Fingerhut A. (1993). Ileocolonic anastomosis after right hemicolectomy for carcinoma: stapled or hand-sewn?. *International Journal of Colorectal Disease*.

[14] Izbicki J. R., Gawad K. A., Quirrenbach S. (1998). Can stapled anastomosis in visceral surgery still be justified? A prospective controlled randomized study of the cost-effectiveness of hand-sewn and stapled anastomoses. *Chirurg*.

[15] Ikeuchi H., Kusunoki M., Yamamura T. (2000). Long-term results of stapled and hand-sewn anastomoses in patients with Crohn's disease. *Digestive Surgery*.

[16] McLeod R. S., Wolff B. G., Ross S., Parkes R., McKenzie M. (2009). Recurrence of Crohn's disease after ileocolic resection is not affected by anastomotic type: results of a multicenter, randomized, controlled trial. *Diseases of the Colon and Rectum*.

[17] Zurbuchen U., Kroesen A. J., Knebel P. (2013). Complications after end-to-end vs. side-to-side anastomosis in ileocecal Crohn's disease—early postoperative results from a randomized controlled multi-center trial (ISRCTN-45665492). *Langenbeck's Archives of Surgery*.

[18] Muñoz-Juárez M., Yamamoto T., Wolff B. G., Keighley M. R. B., Mortensen N. (2001). Wide-lumen stapled anastomosis vs. conventional end-to-end anastomosis in the treatment of Crohn's disease. *Diseases of the Colon and Rectum*.

[19] Mäkelä J. T., Kiviniemi H., Laitinen S. (2003). Risk factors for anastomotic leakage after left-sided colorectal resection with rectal anastomosis. *Diseases of the Colon & Rectum*.

[20] Snijders H. S., Wouters M. W. J. M., van Leersum N. J. (2012). Meta-analysis of the risk for anastomotic leakage, the postoperative mortality caused by leakage in relation to the overall postoperative mortality. *European Journal of Surgical Oncology*.

[21] Walker K. G., Bell S. W., Rickard M. J. F. X. (2004). Anastomotic leakage is predictive of diminished survival after potentially curative resection for colorectal cancer. *Annals of Surgery*.

[22] Golub R., Golub R. W., Cantu R., Stein H. D. (1997). A multivariate analysis of factors contributing to leakage of intestinal anastomoses. *Journal of the American College of Surgeons*.

[23] Kanellos I., Blouhos K., Demetriades H. (2004). The failed intraperitoneal colon anastomosis after colon resection. *Techniques in Coloproctology*.

[24] Simillis C., Purkayastha S., Yamamoto T., Strong S. A., Darzi A. W., Tekkis P. P. (2007). A meta-analysis comparing conventional end-to-end anastomosis vs. other anastomotic configurations after resection in Crohn's disease. *Diseases of the Colon & Rectum*.

[25] Vonlanthen R., Slankamenac K., Breitenstein S. (2011). The impact of complications on costs of major surgical procedures: a cost analysis of 1200 patients. *Annals of Surgery*.

[26] Ruiz-Tovar J., Santos J., Arroyo A. (2012). Microbiological spectrum of the intraperitoneal surface after elective right-sided colon cancer: are there differences in the peritoneal contamination after performing a stapled or a handsewn anastomosis. *International Journal of Colorectal Disease*.

